# Association between serum lipids and low back pain among a middle-aged Japanese population: a large-scale cross-sectional study

**DOI:** 10.1186/s12944-018-0907-1

**Published:** 2018-11-24

**Authors:** Takahiko Yoshimoto, Hirotaka Ochiai, Takako Shirasawa, Satsue Nagahama, Mariko Kobayashi, Akira Minoura, Ayako Miki, Yingli Chen, Hiromi Hoshino, Akatsuki Kokaze

**Affiliations:** 1 Department of Hygiene, Public Health and Preventive Medicine, Showa University School of Medicine, 1-5-8 Hatanodai, Shinagawa-ku, Tokyo 142-8555 Japan; 2 All Japan Labor Welfare Foundation, 6-16-11 Hatanodai, Shinagawa-ku, Tokyo 142-0064 Japan

**Keywords:** Low back pain, Lipid, HDL cholesterol, LDL cholesterol, LDL-C/HDL-C ratio

## Abstract

**Background:**

Abnormal lipid levels have been suggested as a mechanism leading to atherosclerosis of the lumbar vessels, resulting in low back pain (LBP). This study examined whether abnormal lipid levels were associated with LBP among middle-aged adults in Japan.

**Methods:**

The present study included adults between 40 and 64 years old who underwent an annual health checkup. A total of 258,367 eligible participants were analyzed to investigate associations of LBP with low-density lipoprotein cholesterol (LDL-C), high-density lipoprotein cholesterol (HDL-C), and LDL-C/HDL-C ratio. Participants were categorized into two groups according to each of LDL-C, HDL-C, and LDL-C/HDL-C ratio (LDL-C: ≥ 140 vs. < 140 mg/dL; HDL-C: ≥ 40 vs. < 40 mg/dL; LDL-C/HDL-C ratio: ≥ 2.5 vs. < 2.5). Information on LBP was obtained using a self-administered questionnaire. Logistic regression modeling was used to calculate the odds ratio (OR) and 95% confidence interval (CI) for LBP.

**Results:**

The prevalence of LBP was 2.2% in men and 2.1% in women. Multivariable analysis adjusting for age, body mass index, and lifestyle factors found significant associations for HDL-C <  40 mg/dL (OR, 1.34; 95%CI, 1.20–1.48 in men; OR, 1.32; 95%CI, 1.02–1.72 in women) and LDL-C/HDL-C ratio ≥ 2.5 (OR, 1.17; 95%CI, 1.09–1.26 in men; OR, 1.15; 95%CI, 1.03–1.29 in women) with LBP.

**Conclusions:**

Low HDL-C and high LDL-C/HDL-C ratio were significantly associated with LBP in a middle-aged Japanese population. These findings might support the atherosclerosis-LBP hypothesis.

## Background

Low back pain (LBP) is a common musculoskeletal health problem worldwide, and the leading cause of years lived with disability [1]. Moreover, LBP has been linked to considerable socio-economic loss, impairing the health of employees, and reducing work productivity [2, 3]. Effective strategies for prevention of LBP are thus urgently required.

The etiology of LBP is multifactorial, including individual, physical, and psychosocial factors [4]. As one of the mechanisms underlying LBP, it has been suggested that atherosclerosis of the lumbar arteries could reduce the blood supply to the lumbar region, leading to disc degeneration and LBP [5, 6]. LBP has been found more frequently in subjects with missing or narrowed lumbar or sacral arteries [7, 8] or calcification in the abdominal aorta [9]. Considering that LBP could be influenced by arterial degeneration, risk factors for atherosclerosis may also be associated with LBP. Several studies have investigated associations of LBP with high levels of blood cholesterol, which are involved in the development of atherosclerosis [10–13]. However, the results have been inconsistent and mostly from Western countries. Given the ethnic differences in lifestyles and the development of metabolic disorders [14], research targeted at the Japanese population is essential to clarify the risk factors for LBP and to explore effective interventions for preventing LBP in this population.

The aim of the present study was to investigate the association between serum lipids (low-density lipoprotein cholesterol (LDL-C), high-density lipoprotein cholesterol (HDL-C), and LDL-C/HDL-C ratio) and LBP in a Japanese population using large-scale data from health checkups.

## Methods

### Study population

Subjects in the present study were adults between 40 and 64 years old who underwent an annual health checkup during the period from April 2013 to March 2014 and conducted by the All Japan Labor Welfare Foundation, a health checkup center in Japan. Of the total of 310,577 participants in health checkups during this period, 310,498 subjects participated in this study. Of these, we excluded from the present study 7525 participants who took medication for dyslipidemia at the time of the checkup, and 44,606 participants with missing data on variables. As a result, data from 258,367 participants were analyzed. Informed consent for the use of personal information in this study was obtained from each participant. The present study complies with the ethical guidelines of the Declaration of Helsinki, and received approval from the medical ethics committee of Showa University School of Medicine (Approval No. 2407) and the Ethics Committee of the All Japan Labor Welfare Foundation (Approval No. 9-1-0007).

### Measurements

A self-administered questionnaire was distributed to each subject who underwent the health checkup. The subject was asked to complete the questionnaire, which included questions on age, sex, and lifestyle. Question items on lifestyle included smoking status (none, former, current), alcohol intake (none, sometimes, everyday), and physical activity equal to walking at least 60 min/day (yes or no), as information recommended to assess health conditions in health checkups by the Ministry of Health, Labour and Welfare in Japan [15]. LBP was self-reported in the following question: “Do you have LBP under treatment including follow-up?” [16]. Measurements of height and weight were performed by trained staff. Height and weight were measured to the nearest 0.1 cm using a stadiometer, and to the nearest 0.1 kg using a scale, respectively. Body mass index (BMI) was calculated as the weight in kilograms divided by the square of the height in meters. Age was classified into strata of 40–49, 50–59, and 60–64 years [11]. BMI was categorized into three groups of < 18.5, 18.5–24.9, and ≥ 25 kg/m^2^ [17].

A venous blood sample was collected and analyzed at an external laboratory (SRL, Tokyo, Japan) within 24 h of being drawn. LDL-C and HDL-C were determined using a direct method (AU5400; Beckman Coulter, Tokyo, Japan). LDL-C and HDL-C were categorized based on the definitions of dyslipidemia by the Japan Atherosclerosis Society Guidelines [18] as follows: LDL-C, high (≥ 140 mg/dL) or normal (< 140 mg/dL); HDL-C, low (< 40 mg/dL) or normal (≥ 40 mg/dL). LDL-C/HDL-C ratio was categorized according to a previous study [19] as high (≥ 2.5) or low (< 2.5).

### Statistical analysis

We compared the characteristics of participants by sex and by the presence or absence of LBP using Pearson’s chi-square test for categorical variables, and the unpaired t-test for continuous variables. To evaluate the relationship between serum lipids and LBP, logistic regression analysis was performed to calculate odds ratios (ORs) and 95% confidence intervals (CIs) for LBP. In the model, age, BMI, smoking status, alcohol intake, and physical activity, were included to control for potential confounders because such lifestyle factors have been recognized as important factors contributing to LBP [20–22]. Statistical analyses were performed using JMP version 13.0 (SAS Institute Japan, Tokyo, Japan). A value of *p* <  0.05 was considered statistically significant, and all reported *p* values are two sided.

## Results

Mean age of participants in the present study was 50.9 years (standard deviation, 7.2 years), and 65.6% of participants were men. The prevalence of LBP was 2.2% for the overall cohort. Participant characteristics by sex in the present study are shown in Table 1. The prevalence of LBP was 2.2% in men, and 2.1% in women.

**Table 1:** Baseline characteristics of study participants by sex (*n* = 258,367)

	Men (*n* = 169,606)		Women (*n* = 88,761)		*p* value^a^
	n	%	n	%	
Age (years)					
40–49	80,774	47.6	40,029	45.1	< 0.001
50–59	61,948	36.5	34,912	39.3	
60–64	26,884	15.9	13,820	15.6	
Height (cm)	169.1 (6.4)		157.2 (6.3)		< 0.001
Weight (kg)	68.5 (11.6)		55.7 (10.3)		< 0.001
Body mass index (kg/m^2^)					
< 18.5	3783	2.2	6241	7.0	< 0.001
18.5–24.9	110,236	65.0	63,475	71.5	
≥ 25	55,587	32.8	19,045	21.5	
Physical activity (min/day)					
≥ 60	58,183	34.3	27,453	30.9	< 0.001
< 60	111,423	65.7	61,308	69.1	
Smoking status					
None	58,054	34.2	65,547	73.8	< 0.001
Former	31,525	18.6	5884	6.6	
Current	80,027	47.2	17,330	19.5	
Alcohol intake					
None	48,457	28.6	50,406	56.8	< 0.001
Sometimes	48,965	28.9	24,558	27.7	
Everyday	72,184	42.6	13,797	15.5	
Low back pain					
+	3726	2.2	1889	2.1	0.256
–	165,880	97.8	86,872	97.9	
LDL-C (mg/dL)					
Normal (< 140)	119,824	70.6	63,407	71.4	< 0.001
High (≥ 140)	49,782	29.4	25,354	28.6	
HDL-C (mg/dL)					
Normal (≥ 40)	154,412	91.0	86,920	97.9	< 0.001
Low (< 40)	15,194	9.0	1841	2.1	
LDL-C/HDL-C ratio					
Low (< 2.5)	105,067	61.9	70,748	79.7	< 0.001
High (≥ 2.5)	64,539	38.1	18,013	20.3	

Data are presented as number and percentage, or mean (standard deviation)

*LDL-C*: Low-density lipoprotein-cholesterol, *HDL-C*: High-density lipoprotein-cholesterol

^a^ Pearson chi-square test or unpaired t test

Tables 2 and 3 show comparisons of characteristics between participants with and without LBP in men and in women. Among men, proportions of low HDL-C level and high LDL-C/HDL-C ratio were significantly higher in participants with LBP than in those without LBP (Table 2). Proportions of low HDL-C level and high LDL-C level and LDL-C/HDL-C ratio were significantly higher in women with LBP than in women without LBP (Table 3).

**Table 2:** Comparison of characteristics between participants with and without low back pain among men

	Low back pain (+) (*n* = 3726)		Low back pain (−) (*n* = 165,880)		*p* value^a^
Age (years)					
40–49	1478	39.7	79,296	47.8	< 0.001
50–59	1455	39.0	60,493	36.5	
60–64	793	21.3	26,091	15.7	
Height (cm)	169.1 (6.6)		169.1 (6.4)		0.486
Weight (kg)	69.3 (12.0)		68.5 (11.6)		< 0.001
Body mass index (kg/m^2^)					
< 18.5	129	3.5	6029	3.6	< 0.001
18.5–24.9	2261	60.7	105,600	63.7	
≥ 25	1336	35.8	54,251	32.7	
Physical activity (min/day)					
≥ 60	1244	33.4	56,939	34.3	0.233
< 60	2482	66.6	108,941	65.7	
Smoking status					
None	1249	33.5	56,805	34.2	< 0.001
Former	813	21.8	30,712	18.5	
Current	1664	44.7	78,363	47.2	
Alcohol intake					
None	1085	29.1	47,372	42.5	0.054
Sometimes	1010	27.1	47,955	28.9	
Everyday	1631	43.8	70,553	28.6	
LDL-C (mg/dL)					
Normal (< 140)	2605	69.9	117,219	70.7	0.320
High (≥ 140)	1121	30.1	48,661	29.3	
HDL-C (mg/dL)					
Normal (≥ 40)	3294	88.4	151,118	91.1	< 0.001
Low (< 40)	432	11.6	14,762	8.9	
LDL-C/HDL-C ratio					
Low (< 2.5)	2178	58.5	102,889	62.0	< 0.001
High (≥ 2.5)	1548	41.5	62,991	38.0	

Data are presented as number and percentage, or mean (standard deviation)

*LDL-C*: Low-density lipoprotein-cholesterol, *HDL-C*: High-density lipoprotein-cholesterol

^a^Pearson chi-square test or unpaired t test

**Table 3:** Comparison of characteristics between participants with and without low back pain among women

	Low back pain (+) (*n* = 1889)		Low back pain (−) (*n* = 86,872)		*p* value^a^
Age (years)					
40–49	626	33.1	39,403	45.4	< 0.001
50–59	863	45.7	34,049	39.2	
60–64	400	21.2	13,420	15.4	
Height (cm)	156.9 (6.5)		157.2 (6.3)		0.062
Weight (kg)	57.7 (10.8)		55.7 (10.3)		< 0.001
Body mass index (kg/m^2^)					
< 18.5	138	7.3	9474	10.9	< 0.001
18.5–24.9	1200	63.5	58,904	67.8	
≥ 25	551	29.2	18,494	21.3	
Physical activity (min/day)					
≥ 60	643	34.0	26,810	30.9	0.003
< 60	1246	66.0	60,062	69.1	
Smoking status					
None	1327	70.2	64,220	73.9	0.001
Former	149	7.9	5735	6.6	
Current	413	21.9	16,917	19.5	
Alcohol intake					
None	1099	58.2	49,307	56.7	0.250
Sometimes	521	27.6	24,037	27.7	
Everyday	269	14.2	13,528	15.6	
LDL-C (mg/dL)					
Normal (< 140)	1278	67.7	62,129	71.5	< 0.001
High (≥ 140)	611	32.3	24,743	28.5	
HDL-C (mg/dL)					
Normal (≥ 40)	1828	96.8	85,092	98.0	< 0.001
Low (< 40)	61	3.3	1780	2.0	
LDL-C/HDL-C ratio					
Low (< 2.5)	1398	74.0	69,350	79.8	< 0.001
High (≥ 2.5)	491	26.0	17,522	20.2	

Data are presented as number and percentage, or mean (standard deviation)

*LDL-C*: Low-density lipoprotein-cholesterol, *HDL-C*: High-density lipoprotein-cholesterol

^a^ Person chi-square test or unpaid t test

Crude and adjusted ORs of serum lipids for LBP are shown in Table 4. Univariate analysis showed that low HDL-C level and high LDL-C/HDL-C ratio were significantly associated with LBP in both sexes. Even when adjusted for age, BMI, smoking status, alcohol intake, and physical activity in multivariable analysis, associations of HDL-C (OR, 1.34; 95%CI, 1.20–1.48 in men; OR, 1.32; 95%CI, 1.02–1.72 in women) and LDL-C/HDL-C ratio (OR, 1.17; 95%CI, 1.09–1.26 in men; OR, 1.15; 95%CI, 1.03–1.29 in women) with LBP remained significant.

**Table 4:** Crude and adjusted odds ratios of serum lipids for low back pain by sex

	Total	Low back pain	Crude		Adjusted^a^	
	N	n (%)	OR	95%CI	OR	95%CI
Men						
LDL-C (mg/dL)						
Normal (< 140)	119,824	2605 (2.2)	1.00		1.00	
High (≥ 140)	49,782	1121 (2.3)	1.04	0.97–1.11	1.04	0.96–1.11
HDL-C (mg/dL)						
Normal (≥ 40)	154,412	3294 (2.1)	1.00		1.00	
Low (< 40)	15,194	432 (2.8)	1.34	1.21–1.49	1.34	1.20–1.48
LDL-C/HDL-C ratio						
Low (< 2.5)	105,067	2178 (2.1)	1.00		1.00	
High (≥ 2.5)	64,539	1548 (2.4)	1.16	1.09–1.24	1.17	1.09–1.26
Women						
LDL-C (mg/dL)						
Normal (< 140)	63,407	1278 (2.0)	1.00		1.00	
High (≥ 140)	25,354	611 (2.4)	1.20	1.09–1.32	1.02	0.92–1.13
HDL-C (mg/dL)						
Normal (≥ 40)	86,920	1828 (2.1)	1.00		1.00	
Low (< 40)	1841	61 (3.3)	1.59	1.23–2.07	1.32	1.02–1.72
LDL-C/HDL-C ratio						
Low (< 2.5)	70,748	1398 (2.0)	1.00		1.00	
High (≥ 2.5)	18,013	491 (2.7)	1.39	1.25–1.54	1.15	1.03–1.29

*OR*: Odds ratio, *CI*: Confidence interval, *LDL-C*: Low-density lipoprotein-cholesterol, *HDL-C*: High-density lipoprotein-cholesterol

^a^ Adjusted for age, body mass index, smoking status, alcohol intake, and physical activity

## Discussion

This study investigated the association between serum lipids and LBP using large-scale data from annual health checkups in Japan. Our results showed that low HDL-C level and high LDL-C/HDL-C ratio were significantly associated with LBP after adjusting for potential confounders. To the best of our knowledge, this represents the first study to demonstrate significant associations between serum lipids and LBP in middle-aged Japanese adults.

Our study indicated that prevalence of LBP under treatment was 2.2% in total participants. Myojin et al. have reported that the prevalence of backache under treatment was 5.25% based on the Comprehensive Survey of Living Conditions, collected by the Ministry of Health, Labour and Welfare of Japan, and with participants including elderly individuals [16]. Considering that older people show a higher prevalence of LBP [23], it may be reasonable for the prevalence of LBP to be lower in our study. In a previous study with more than 5000 health examinees [24], prevalence of LBP treated among 40–59 years old was 3.2%, similar to our results.

The present study found a significant association between low HDL-C level and LBP. Low HDL-C level is considered as an independent risk factor for cardiovascular events [25, 26]. Heuch et al. found that the prevalence of LBP was inversely associated with HDL-C among women in a nationally representative sample study [11]. Moreover, in a cohort study, low HDL-C level could represent a risk factor for chronicity of LBP in men with LBP at baseline [27]. Such results suggest that our study findings in a Japanese population were reasonable.

A mechanism by which abnormality of serum lipid concentrations causes LBP might be explained through atherosclerosis of the involved arteries; the atherosclerosis obstructs blood supply to corresponding lumbar region, resulting in disc degeneration and damage to surrounding tissues [5, 28]. Several studies have indicated more frequent LBP in patients with various lesions in the arteries involved [7, 8, 29]. Because disorders of lipid metabolism are considered essential for initiating the long, drawn-out process of atherosclerosis development, our results are in accordance with the hypothesis that atherosclerosis of the lumbar vessels is significant as a mechanism leading to LBP. Although lipid levels may indirectly affect LBP through obesity, which has been considered to result in increased mechanical load on the lumbar structure [30], our results showed a significant relationship between LBP and lipid levels after adjustment of BMI. These results may imply the existence of another pathway contributing to the development of LBP other than obesity. Another mechanism might involve inflammation. Pro-inflammatory cytokines have been shown to influence lipid metabolism via stimulating fatty acid synthesis or lipolysis [31]. Chronic LBP patients have been shown to have higher levels of pro-inflammatory cytokines than those without LBP [32, 33]. Because information on pro-inflammatory cytokines was not obtained in our study, future studies will be needed to elucidate the mechanisms involved.

In the present study, a high LDL-C/HDL-C ratio was significantly associated with LBP. LDL-C/HDL-C ratio has recently gained attention as an index for cardiovascular disease risk [19, 34]. This study used a cutoff for LDL-C/HDL-C ratio of 2.5 according to the previous study related to cardiovascular risk [19]. The cutoff value of 2.5 is consistent with the value from an intravascular ultrasonographic study that indicated a drastic increase in plaque formation in the coronary artery [35]. In addition, as an optimal reference value for cholesterol, the National Cholesterol Education Program guidelines recommend levels of LDL-C and HDL-C that represent a ratio of about 2.5 [36]. Our results suggest that LDL-C/HDL-C ratio ≥ 2.5, which can be obtained from a standard lipid profile, may be useful as a marker to detect the risk of LBP from the standpoint of the atherosclerosis-LBP hypothesis.

A major strength in the present study was that the subject population was a large-scale sample in Japan. In contrast, our study has some limitations. First, information on LBP was obtained from a self-reported questionnaire in the health checkup, and was not based on specific clinical examinations or a disease-specific questionnaire. Distinguishing between localized pain and radicular pain, or acute and chronic pain was thus not possible. However, such assessments of LBP in detail may be difficult to achieve in large population-based research. Second, confounding by unmeasured variables such as occupation, psychosocial characteristics, or unhealthy lifestyles including sedentary behavior or sleep disturbance [37–39] cannot be ruled out. These factors could contribute to both LBP and dyslipidemia without causal relationship between them. Finally, the direction of causality of the relationship was not able to be inferred because of the cross-sectional design used in this study. For example, it cannot deny the possibility that LBP would restrict physical activity over time which lead to dyslipidemia. Further longitudinal research is needed to clarify the causal relationship.

## Conclusions

In conclusion, a low HDL-C level and high LDL-C/HDL-C ratio were significantly associated with LBP in a middle-aged Japanese population. The present study may have important implications for elucidation of the pathophysiological mechanisms of LBP.

## Abbreviations

BMI: Body mass index
CI: Confidence interval
HDL-C: High-density lipoprotein-cholesterol
LBP: Low back pain
LDL-C: Low-density lipoprotein-cholesterol
OR: Odds ratio
